# Prognostic Role of Serum Lactate Dehydrogenase in Patients With Urothelial Carcinoma: A Systematic Review and Meta-Analysis

**DOI:** 10.3389/fonc.2020.00677

**Published:** 2020-05-20

**Authors:** Minhong Wu, Pengxiu Lin, Lifang Xu, Zhiling Yu, Qingsheng Chen, Hongyong Gu, Cailing Liu

**Affiliations:** ^1^Department of Urology, Yichun People's Hospital, Yichun, China; ^2^Department of Medical Record Management, Chinese Air Force Specialty Medical Center, Beijing, China

**Keywords:** urothelial carcinoma, lactate dehydrogenase, prognosis, systematic review, meta-analysis

## Abstract

**Background:** To investigate the potential prognostic role of serum lactate dehydrogenase (LDH) in patients with urothelial carcinoma (UC) using the method of systematic review and meta-analysis.

**Materials and Methods:** We searched PubMed, Embase, Cochrane Library, and Web of Science for eligible studies up to February 2020. Pooled hazard ratios (HRs) and 95% confidence intervals (CIs) were used to estimate the relationship.

**Results:** A total of 14 studies including 4,009 patients with UC were incorporated. The results showed that a high pretreatment serum LDH was associated with an inferior overall survival (OS, HR 1.61, 95% CI 1.39–1.87, *p* < 0.001), cancer-specific survival (CSS, HR 1.41, 95% CI 1.05–1.90, *p* = 0.022), and disease-free survival (DFS, HR 1.64, 95% CI 1.04–2.59, *p* = 0.034) in UC. Subgroup analyses identified that a high pretreatment serum LDH was associated with a poor OS (HR 1.97, 95% CI 1.02–3.81, *p* = 0.042) and DFS (HR 1.64, 95% CI 1.04–2.59, *p* = 0.034) in upper tract urothelial carcinoma, a short OS (HR 1.71, 95% CI 1.37–2.15, *p* < 0.001) in urothelial carcinoma of bladder.

**Conclusion:** Our findings indicated that a high level of pretreatment serum LDH was associated with inferior OS, CSS, and DFS in patients with UC. This biomarker can be an important factor incorporated into the prognostic models for UC.

## Introduction

Urothelial carcinoma (UC), mainly consisting of upper tract urothelial carcinoma (UTUC) and urothelial carcinoma of the bladder (UCB), is, respectively estimated to have 85,000 new incidences and 18,000 related mortality in the United States in 2018. These made UC become the 4th and 12th most common malignance for males and females (1). Multiple lesions, high rate of recurrences, and distant metastasis are typical features of UC. Despite advances in surgical techniques and developments of preoperative and postoperative adjuvant therapy, the long-term survival of cases with UC have not significantly changed over these years (2). Especially, for patients with advanced UC, median overall survival was only 3 to 6 months without therapy, and prolonged to 13–16 months when receiving systematic chemotherapy (3). Therefore, it is important to determine prognostic factors for timely adjustment of treatment.

Previous literatures have found that cancer cells metabolize differently from normal cells, which means more lactate seems to be needed for cancer cells (4). Lactate dehydrogenase (LDH), an enzyme that catalyzes lactic acid into pyruvate, may exert a crucial role in the metabolism of tumor cells. High level of serum LDH has been reported to serve as an unfavorable prognostic factor in kinds of malignances, including prostate cancer, renal cell carcinoma, lymphoma, colorectal cancer, and lung cancer (5). Moreover, many studies have identified the prognostic role of pretreatment serum LDH in cases with UC. Because circulating blood LDH is easy to be measured clinically, it can be used as an indicator of cancer burden and a useful biomarker in clinical management. In the present study, we aimed to systematic review literatures studying the prognostic role of pretreatment LDH in UC and merged the quantitative data.

## Methods

### Study Design

The present study was performed according to the PRISMA statements (Supplementary Material) (6). The protocol has been registered on PROSPERO (No. CRD42019147216).

### Literature Searching

In order to examine the prognostic significance of serum lactate dehydrogenase in urothelial carcinoma, we searched databases PubMed, Embase, Cochrane Library, and Web of Science up to February 2020 to identify related literatures. The following major terms were used to constitute the search strategy: “lactate dehydrogenase” (e.g., “lactate dehydrogenase,” “LDH,” “lactic dehydrogenase”), “urothelial carcinoma” (e.g., “urothelial carcinoma,” “transitional cell carcinoma,” “urothelial tumor,” “urothelial cancer”), and “prognosis” (e.g., “prognosis,” “survival,” “progression,” “recurrence,” “mortality,” “outcome”). Additionally, we manually screened literature references to identify relevant studies. There was no language restriction in the process of literature searching.

### Selection Criteria

In general, the present study included literatures investigating the prognostic role of pretreatment of serum lactate dehydrogenase (LDH) in patients with urothelial carcinoma, and detailed criteria were shown as follows. Inclusion criteria: (1) studies involving patients confirmed diagnosed with urothelial carcinoma; (2) studies that measured serum lactate dehydrogenase of patients before treatment; (3) studies that reported the results of oncological outcomes including overall survival (OS), cancer-specific survival (CSS), and disease-free survival (DFS); (4) studies that directly provided hazard ratios (HRs) and 95% confidence interval (CI) or required data for calculating them. The methods for calculation were reported by Tierney et al. (7). Exclusion criteria: (1) studies wherein we cannot extract HRs and 95% CI; (2) studies that did not analyze serum lactate dehydrogenase as dichotomous variable or did not clearly report the cut-off value; (3) not original article, such as review, abstract, opinion, letter, and so on; (4) duplicated results from the same cohort. Two researchers independently screened and evaluated the literature; the disputes were resolved by discussion.

### Data Extraction and Quality Assessment

These two important steps were independently performed by two researchers, and the disputes were resolved by discussion. According to a pre-designed table, items of data extraction included the first author's last name, publication year, belonging country, number of subjects included, patient's age, cancer type (urothelial carcinoma, upper tract urothelial carcinoma, or urothelial carcinoma of bladder), cancer stage, cut-off value and decision method, therapies that patients underwent, endpoints of oncological outcomes, HRs and 95% CIs (from univariate or multivariate Cox analysis), follow-up durations, and adjusted variables in multivariate Cox analysis.

The quality of each study was evaluated using the Newcastle–Ottawa scale, which embraced three aspects including patient selection, comparability, assessment of outcome, and follow-ups.

### Statistical Analysis

We extracted all HRs and 95% CIs of related endpoints from included studies. Subgroup analyses of overall survival were also conducted. The grouping variables included publication year, region, site of malignance, number of patients, cancer stage, cut-off value, and NOS score. Meta-regression was also performed to identify the resources of interstudy heterogeneity. Subgroup analyses of upper tract urothelial carcinoma (UTUC) and urothelial carcinoma of the bladder (UCB) were also performed. HRs and the corresponding 95% CI were used to assess the significance of the prognostic value of serum lactate dehydrogenase for urothelial carcinoma. A pooled value >1 was regarded as indicating an unfavorable outcome for the subjects having a high level of serum lactate dehydrogenase. Cochran's Q test and Higgins *I*^2^ statistic were both conducted to examine inter-study heterogeneity. A random effects model was applied for all analyses. Publication bias was assessed by funnel plots, Egger's, and Begg's tests. Sensitivity analysis was performed to check the stability of the results. All statistical analyses were performed with Stata 12.0 (STATA Corporation, College Station, TX, USA).

## Results

### Study Searching and Screening

**Figure 1:**
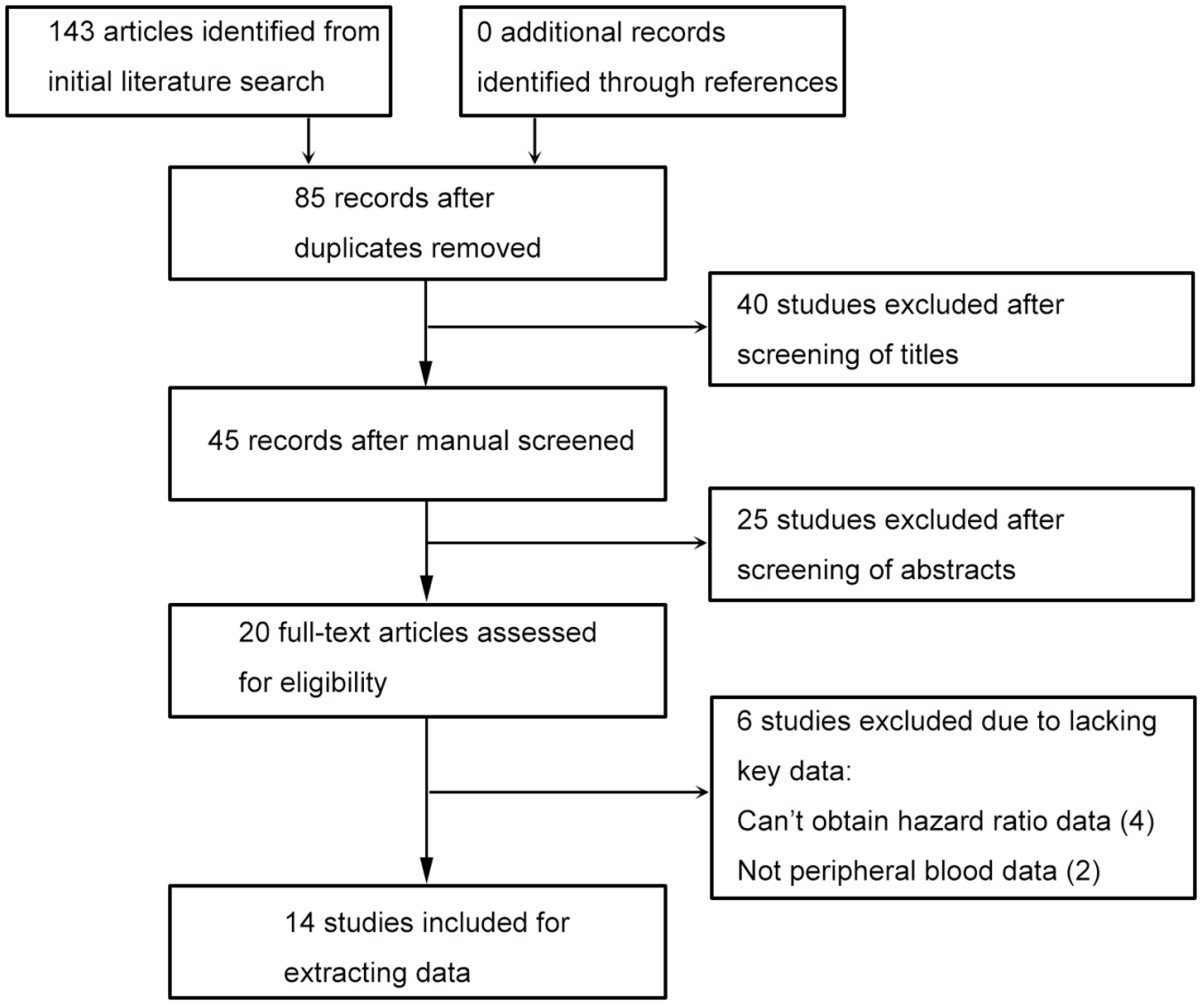
Flowchart of study searching and screening.

The flowchart of this process is presented in (Figure 1). Database searching identified 143 articles, and no additional study was found by checking the references. Eighty-five studies were remained after excluding duplicated records. Title and abstract screening, respectively, excluded 40 and 25 literatures, then 20 studies were assessed with full text. Since there were four studies wherein hazard ratio data cannot be obtained (8–11), and two studies had no peripheral blood date (12, 13), 14 literatures were included last for data extraction (2, 14–25).

Baseline features of the included studies are shown in (Table 1). Nine studies were published recently (2013–2020) by Chinese and Japanese researchers (2, 14–20, 26). Five studies were published 16 years (1993–2002) ago by Chinese and European researchers (21–25). The median or mean age of patients ranged from 60.3 to 73 years, and the sample size ranged from 56 to 1,087. Seven studies focused on urothelial carcinoma (14, 16, 19, 22–24, 26), three focused on UTUC (2, 17, 18), and four focused on UCB (15, 20, 21, 25). Of all 40 studies, 10 provided results of multivariable Cox analysis (2, 14, 15, 17–20, 22, 25, 26). The adjusted factors embraced clinical, pathological, and laboratorial variables, which are detailed in (Table 2). All studies were of low-to-moderate risk of bias, the NOS score ranged from 6 to 8, which are presented in (Table 3).

**Table 1:** Baseline features of included studies.

**References**	**Country**	**Sample size**	**Age (years)**	**Cancer type**	**Cancer stage**	**Cut-off value (U/l)**	**Decision method**	**Therapy**	**Survival analysis**
Suzuki et al. (26)	Japan	185	70 (64–76)	UC	Advanced	360	LRM	All	Multivariate
Takemura et al. (19)	Japan	125	70 (64–74)	UC	Advanced	246	Reported	All	Multivariate
Tan et al. (2)	China	668	65.8 ± 11.4	UTUC	All	220	Normal	All	Multivariate
Nakagawa et al. (15)	Japan	1087	69 (63–75)	UCB	Recurrent	ULN	Normal	All	Multivariate
Abe et al. (16)	Japan	228	67 (30–83) R	UC	Metastatic	200	-	Chemotherapy	Univariate
Zhang et al. (17)	China	100	60.3 mean	UTUC	Localized	245	Normal	Surgery	Multivariate
Ito et al. (18)	Japan	71	-	UTUC	Localized	210	Normal	Surgery	Multivariate
Fukushima et al. (19)	Japan	88	68 (39–91)R	UC	Advanced	ULN	Normal	All	Multivariate
Nakagawa et al. (20)	Japan	114	67(32–84)R	UCB	Recurrent	ULN	Normal	All	Multivariate
Yang et al. (21)	China	310	-	UCB	Advanced	200	Reported	Surgery and chemotherapy	Univariate
Yang et al. (22)	China	535	71 (31–88) R	UC	All	200	Reported	All	Multivariate
Bellmunt et al. (23)	Spain	56	-	UC	Advanced	ULN	Normal	Chemotherapy	Univariate
Sengelov et al. (24)	Denmark	240	67 (37–86)R	UC	Recurrent or metastatic	ULN	Normal	Surgery and chemotherapy	Univariate
Hannisdal et al. (25)	Norway	202	73 (32–85)R	UCB	Localized	400	-	Radiotherapy	Multivariate

*UC, urothelial carcinoma; UTUC, upper tract urothelial carcinoma; UCB, urothelial carcinoma of the bladder; ULN, upper limit of normal; LRM, linear regression model; R, range*.

**Table 2:** Follow-up and oncological outcomes.

**References**	**Follow-up duration, month**	**Outcomes**	**Adjusted factors**
Suzuki et al. (26)	12.3	OS	Age, performance status, BMI, primary tumor site, alkaline phosphatase, C-reactive protein, albumin, lymphocyte count, Pembrolizumab after chemotherapy failure
Takemura et al. (19)	12.1	OS	Age, Karnofsky performance status, primary site, neutrophil-to-lymphocyte ratio, γ-glutamyltransferase, C-reactive protein, systemic chemotherapy
Tan et al. (2)	45 (21–74)	OS, CSS, DFS	Tumor grade, concomitant variant histology, lymphovascular invasion, tumor size, tumor architecture, surgical margin status, perioperative blood transfusion, anemia, pT stage, lymph node status, alpha-hydroxybutyrate dehydrogenase, alkaline phosphatase, albumin, globulin, white blood cells, adjuvant therapy
Nakagawa et al. (15)	6.8 (3.0–15.8)	OS	Age, pT stage, lymph node density, time-to-recurrence, symptom, number of involved organs/sites, local recurrence, bone metastasis, liver metastasis, lung metastasis, hemoglobin level, leukocyte count, platelet count, total protein, albumin, alkaline phosphatase, C-reactive protein, estimated glomerular filtration rate, treatments after recurrence, era of recurrence
Abe et al. (16)	17 (14–19)	OS	-
Zhang et al. (17)	45.8 (1–151)R	OS, DFS	Pathological stage, lymph node status, subsequent bladder tumor, tumor grade, multifocality, vascular invasion, tumor necrosis, architecture
Ito et al. (18)	50.3 (1–160)R	DFS	Clinical T stage
Fukushima et al. (19)	13 (1–99)R	OS	Primary site, alkaline phosphatase, C-reactive protein, sarcopenia
Nakagawa et al. (20)	11.0 (0.2–206.7)R	OS	Time-to-recurrence, symptoms at recurrence, no metastatic organs, C-reactive protein, post-recurrent chemotherapy with platinum agent, metastasectomy
Yang et al. (21)	71 (1–132)R	CSS	-
Yang et al. (22)	81 (1–144)R	OS	Urbanization, from endemic “blackfoot disease” area, age, sex, histologic grade, T stage, lymph node metastases, distant metastases, serum creatinine level
Bellmunt et al. (23)	15.8 (11.9–19.5)	OS	-
Sengelov et al. (24)	3.6 (0.1–62.4)R	OS	-
Hannisdal et al. (25)	18	OS	T stage, ESR, age, albumin

*OS, overall survival; CSS, cancer-specific survival; DFS, disease-free survival; BMI, body mass index; ESR, erythrocyte sedimentation rate; R, range*.

**Table 3:** Newcastle–Ottawa scale for risk of bias assessment.

**References**	**Selection**				**Comparability**	**Outcome**			**Overall**
	**Representativeness of exposed cohort**	**Selection of nonexposed**	**Ascertainment of exposure**	**Outcome not present at start**		**Assessment of outcome**	**Adequate follow-up length**	**Adequacy of follow-up**	
Suzuki et al. (26)	1	1	1	1	1	1	1	1	**8**
Takemura et al. (19)	0	1	1	1	1	1	1	1	**7**
Tan et al. (2)	1	1	1	1	1	1	0	1	**7**
Nakagawa et al. (15)	1	1	1	1	1	1	1	1	**8**
Abe et al. (16)	1	1	1	1	0	1	1	1	**7**
Zhang et al. (17)	0	1	1	1	1	1	0	1	**6**
Ito et al. (18)	0	1	1	1	1	1	0	1	**6**
Fukushima et al. (19)	0	1	1	1	1	1	1	1	**7**
Nakagawa et al. (20)	0	1	1	1	1	1	1	1	**7**
Yang et al. (21)	1	1	1	1	0	1	1	0	**6**
Yang et al. (22)	1	1	1	1	1	1	1	1	**8**
Bellmunt et al. (23)	0	1	1	1	0	1	1	1	**6**
Sengelov et al. (24)	1	1	1	1	0	1	1	1	**7**
Hannisdal et al. (25)	1	1	1	1	1	1	0	1	**7**

### Urothelial Carcinoma

**Figure 2:**
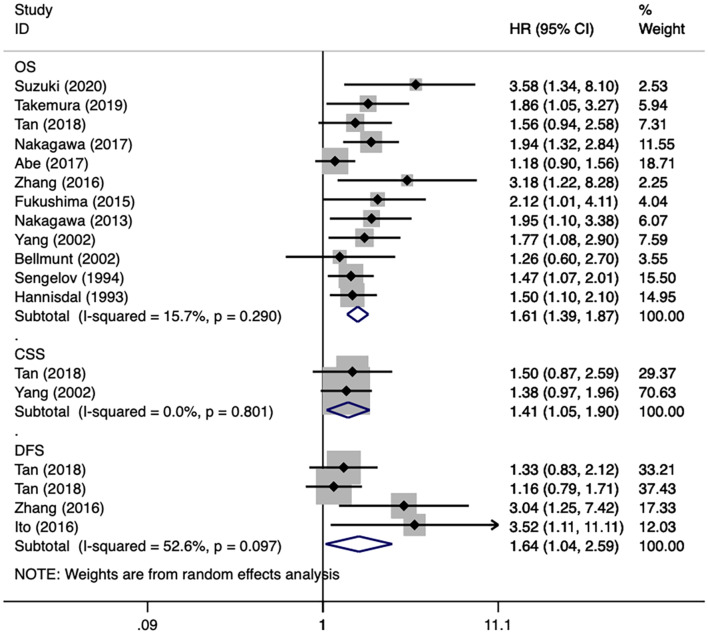
Forest plots of studies about urothelial carcinoma.

First, all included studies were analyzed together. For the three endpoints (overall survival, cancer-specific survival, disease-free survival), there were 12 (2, 14–17, 19, 20, 22–26), 2 (2, 21), and 3 (2, 17, 18) studies, respectively, that reported related results. After merging HRs and 95% CIs, we identified that a high pretreatment serum LDH was associated with an inferior overall survival (HR 1.61, 95% CI 1.39–1.87, *p* < 0.001), cancer-specific survival (HR 1.41, 95% CI 1.05–1.90, *p* = 0.022), and disease-free survival (HR 1.64, 95% CI 1.04–2.59, *p* = 0.034) in patients with urothelial carcinoma (Figure 2). Subgroup analyses of overall survival were also conducted, and the results are presented in (Table 4). The subgroup variables included year of publication, region, site of carcinoma, sample size, survival analysis, and NOS score, which did not obviously change the results. Moreover, except for survival analysis (*p* = 0.033), other subgroup variables were not the resources of inter-study heterogeneity (*P* > 0.05 for all).

**Table 4:** Subgroup analyses for overall survival in urothelial carcinoma.

**Subgroup**	**Studies**	**HR (95% CI)**	***P* value**	**Meta-regression *P* value**	**Heterogeneity**	
					***I^**2**^* (%)**	**P value**
Year of publication				0.845		
2016–2020	6	1.78 (1.31–2.43)	<0.001		54.0	0.054
1993–2015	6	1.59 (1.33–1.90)	<0.001		0.0	0.846
Region				0.350		
Asia	9	1.64 (1.40–1.93)	<0.001		33.7	0.148
Non-Asia	3	1.47 (1.18–1.83)	<0.001		0.0	0.911
Site of carcinoma				0.355		
Upper urinary tract	2	1.82 (1.16–2.84)	0.009		41.3	0.192
Bladder	3	1.71 (1.37–2.15)	<0.001		0.0	0.548
All	7	1.48 (1.25–1.75)	<0.001		29.8	0.201
Sample size				0.084		
> 200	6	1.48 (1.28–1.71)	<0.001		0.0	0.425
<200	6	2.04 (1.53–2.71)	<0.001		0.0	0.537
Survival analysis				0.033		
Multivariable	9	1.82 (1.53–2.15)	<0.001		0.0	0.695
Univariate	3	1.30 (1.06–1.59)	0.010		0.0	0.586
NOS score				0.106		
>7	3	2.00 (1.50–2.66)	<0.001		0.0	0.386
< =7	9	1.49 (1.29–1.72)	<0.001		0.0	0.457

*HR, hazard ratio; CI, confidence interval; NOS, Newcastle–Ottawa Scale*.

### UTUC and UCB

**Figure 3:**
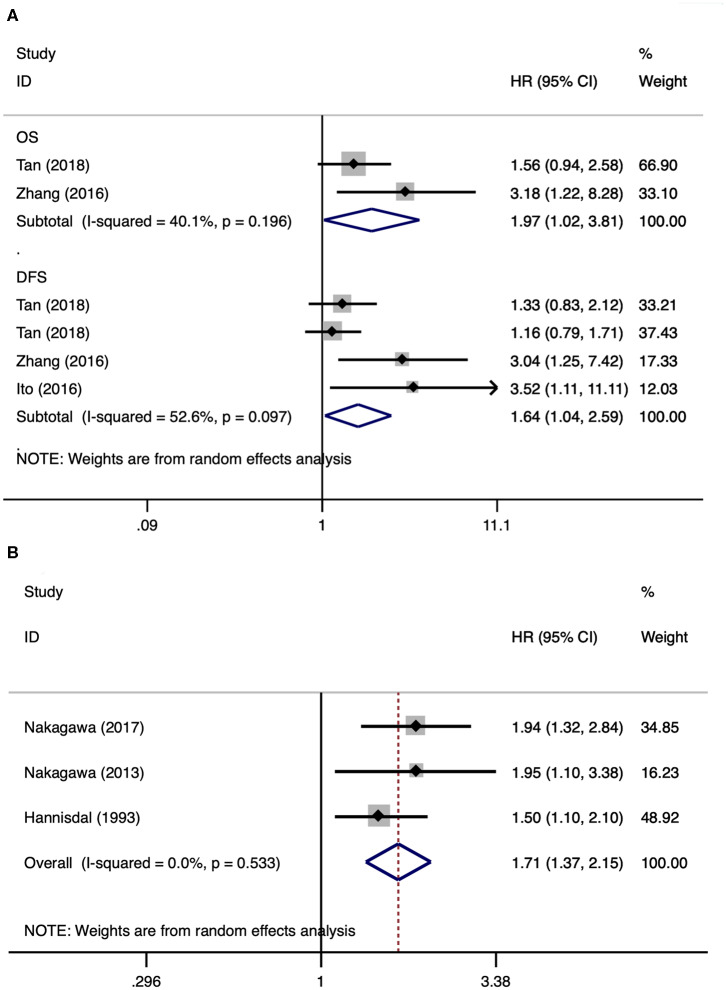
Forest plots of studies about upper tract urothelial carcinoma and urothelial carcinoma of the bladder. **(A)** Upper tract urothelial carcinoma; **(B)** urothelial carcinoma of the bladder.

Subgroup analyses of UTUC and UCB were also performed. There were three and three studies, respectively, that were included in the subgroup analyses of UTUC (2, 17, 18) and UCB (15, 20, 25). After merging HRs and 95% CIs, we identified that a high pretreatment serum LDH was associated with a poor overall survival (HR 1.97, 95% CI 1.02–3.81, *p* = 0.042) and disease-free survival (HR 1.64, 95% CI 1.04–2.59, *p* = 0.034) in patients with UTUC (Figure 3A). A high pretreatment serum LDH was associated with a short overall survival (HR 1.71, 95% CI 1.37–2.15, *p* < 0.001) in patients with UCB (Figure 3B).

### Publication Bias and Sensitivity Analysis

**Figure 4:**
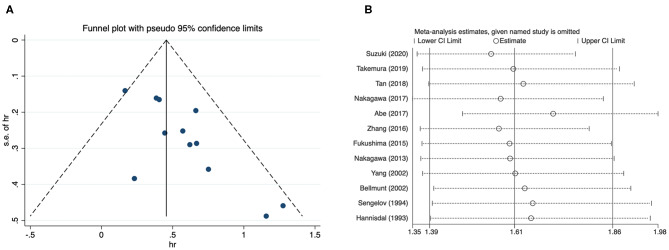
Funnel plot and sensitivity analysis for overall survival of urothelial carcinoma. **(A)** Funnel plot; **(B)** sensitivity analysis.

Since most analyses embraced insufficient literatures, we only checked publication bias and performed sensitivity analysis for overall survival of patients with urothelial carcinoma. The funnel plot showed an approximately asymmetric result, and quantitative tests identified significant differences (Begg's test: *p* = 0.024; Egger's test: *p* = 0.005) (Figure 4A). The result of sensitivity analysis showed that excluding any study did not significantly change the merged data (Figure 4B).

## Discussion

Previous literatures have found that cancer cells metabolize differently from normal cells. Even with sufficient oxygen, malignant cells will preferentially metabolize glucose through glycolysis to produce adequate energy for growth, which is known as the Warburg effect and is one of the major metabolic changes in the process of malignant transformation (4). The serum level of LDH, the enzyme involved in the glycolytic pathway, can reflect the metabolic alterations (27). A high level of serum LDH has been reported to serve as an unfavorable prognostic factor in several types of malignances. Several studies have examined the prognostic role of LDH in subjects with UC and have reported inconsistent results. Hence, the present systematic review and meta-analysis about this issue was performed.

After systematic literature searching and screening, 14 studies embracing 4,009 patients were included. All studies were of low-to-moderate risk of bias, and the NOS score ranged from 6 to 8. The HRs and 95% CIs extracted from these studies were merged. The results showed that a high pretreatment serum LDH was associated with an inferior overall survival, cancer-specific survival, and disease-free survival in UC. Subgroup analyses of OS showed that grouping variable did not obviously change the results. Moreover, sensitivity analysis further confirmed the stability and reliability of the results. Subgroup analyses of UTUC and UCB were also performed. We identified that a high pretreatment serum LDH was associated with a poor overall survival and disease-free survival in UTUC, a short overall survival in UCB. Since circulating blood LDH is easy to be measured clinically, it can be used as an indicator of cancer burden and a useful biomarker in management of UC.

For the prognostic significance of pretreatment LDH in cases with UC, only one meta-analysis previously was reported. In 2016, Zhang et al. (28) performed a meta-analysis to evaluate the prognostic role of LDH for cases with urological cancer. They included two studies about bladder cancer and one study about UTUC. A high level of serum LDH has been reported to serve as an unfavorable prognostic factor. With more and later studies being included, the present study may provide more comprehensive and reliable results for this issue.

Metabolically, the most prominent feature of cancer cells is an increase in lactic acid production due to their increased glucose uptake rate and reduced oxidative phosphorylation rate, regardless of the availability of oxygen. The phenomenon, called aerobic glycolysis, was first discovered 70 years ago by Otto Warburg (4). As an important substance of the Warburg effect, LDH exists in nearly all kinds of tissues, which has six different isoenzymes. These isoenzymes are assembled from two protein subunits, LDHA and LDHB, into a homo- or hetero-tetramer structure (29). The third protein subunit, called LDHC, forms the testicular specific subtype LDH-6 (30). The main function of lactate dehydrogenase is to catalyze the reversible reaction between pyruvate and lactic acid. NAD^+^ is produced along with this process and is necessary for the continuous production of ATP to maintain glycolysis. Among the LDH isoforms, LDH-5 has the highest catalytic efficiency (31). When cancer tissue is necrotic, high intracellular LDH levels are released into the blood, increasing serum LDH concentrations (32). In addition, when distant metastases occur, tumor cells can damage adjacent organs, such as the lung, liver, and bone. Damage to these organs can also increase serum LDH levels (31, 33–35). In conclusion, serum LDH seems to be a significant factor in the development of malignance. Its level can reflect tumor burden and serve as cancer biomarker.

There were several limitations for the present study. First, due to limited literatures, we studied all types and stages of UC together. The differences in type, stage, and treatment for UC may be the sources of inter-study heterogeneity. Despite both belonging to UC, UTUC, and UCB may have different biological behaviors and disease characteristics. Hence, subgroup analyses of UTUC and UCB were also performed. Second, serum LDH may be affected by other non-tumor diseases, such as anemia, hepatic disease, muscular dystrophy, and heart failure, which have not been examined in these studies. Moreover, the present study also included results from univariable analysis. These uncontrolled factors may affect the results, and the methods of survival analysis have been found to be the source of inter-study heterogeneity. Third, taken that different cut-off values were used in the included literatures, it is a problem to choose the best one, which may affect the application of this biomarker in clinical practice. Fourth, a significant publication bias was found in the meta-analysis of overall survival, which cannot be overcome by statistical methods. Regardless, the present study represents the most comprehensive and latest systematic review and meta-analysis about the prognostic role of LDH in patients with UC.

In general, the present study proved that a high level of pretreatment serum LDH was associated with inferior OS, CSS, and DFS in patients with UC. Moreover, an elevated level of serum LDH was associated with poor OS and DFS in patients with UTUC, a short OS in patients with UCB. Despite more high-level studies were needed to verify our results, the level of serum LDH can be an important factor incorporated into the prognostic models for UC.

## Author Contributions

MW and PL: protocol/project development. LX, ZY, and QC: data collection or management. MW, HG, and CL: data analysis. MW and PL: manuscript writing/editing. All authors read and approved the final manuscript.

## Conflict of Interest

The authors declare that the research was conducted in the absence of any commercial or financial relationships that could be construed as a potential conflict of interest.
